# Assessment of agreement and interchangeability between the TEG5000 and TEG6S thromboelastography haemostasis analysers: a prospective validation study

**DOI:** 10.1186/s12871-019-0717-7

**Published:** 2019-03-30

**Authors:** P. Lloyd-Donald, L. Churilov, F. Zia, R. Bellomo, G. Hart, P. McCall, J. Mårtensson, N. Glassford, L. Weinberg

**Affiliations:** 1 Department of Anaesthesia, Austin Hospital, 145 Studley Rd, Heidelberg, Victoria 3084 Australia; 2 Melbourne Brain Centre, 245 Burgundy St, Heidelberg, Victoria 3084 Australia; 3 Department of Intensive Care, Austin Hospital, 145 Studley Rd, Heidelberg, Victoria 3084 Australia; 4 Department of Anaesthesia and Intensive Care Medicine, Karolinska University Hospital, Solna, Sweden; 5 Department of Physiology and Pharmacology, Karolinska Institutet, SE-171 77 Stockholm, Sweden

**Keywords:** Coagulation, Haemostasis, Thromboelastography, Viscoelastic, Device, Monitoring

## Abstract

**Background:**

TEG6S**®** and TEG5000**®** (Haemonetics Corp, USA) are haemostasis analysers that measure viscoelasticity properties of whole blood. Both use different mechanisms to assess similar components of the coagulation process. The aim of this study was to assess agreement and interchangeability between the TEG6S and TEG5000 analysers.

**Methods:**

3.5 mL whole blood was collected from 25 adult patients in a tertiary intensive care unit (ICU). Analysis was performed using TEG6S and TEG5000 haemostatic platforms. Agreement between platforms was measured using Lin’s concordance coefficient (Lin’s CC), further validated using intraclass correlation coefficients and reduced major axis regression (RMAR).

**Results:**

Sixteen (64%) patients were male; mean (range) age: 59yo (23–86). TEG6S and TEG5000 systems were broadly interchangeable. The majority of TEG variables demonstrated almost perfect or substantial agreement and minimal proportional bias (maximum amplitude demonstrated a fixed bias). LY30%, however, demonstrated poor agreement and a proportional bias. Lin’s CC coefficients (95% CI, RMAR slope, intercept) between TEG6S and TEG5000 variables were: R time: 0.78 (0.64–0.92, 0.76, 0.92); K time: 0.82 (0.69–0.94, 1.30, − 0.93); alpha angle: 0.79 (0.64–0.95, 1.04, − 1.43); maximum amplitude (MA): 0.90 (0.83–0.96, 0.99, − 5.0); LY30%: 0.34 (0.1–0.58, 0.43, 0.04).

**Conclusions:**

Adult patients with critical illness demonstrate almost perfect agreement in the R time and MA, substantial agreement in K time and alpha angle, but poor agreement in LY30%, as measured by the TEG6S and TEG5000 analysers. With the exception of LY30%, the TEG6S and TEG5000 platforms appear interchangeable. This has important implications for use in clinical practice and multi-site research programs.

**Trial registration:**

ANZCRT number: 12617000062325, registered 12/Jan17. Retrospectively registered.

**Electronic supplementary material:**

The online version of this article (10.1186/s12871-019-0717-7) contains supplementary material, which is available to authorized users.

## Background

Thromboelastography (TEG) allows rapid, comprehensive and accurate identification of an individual’s haemostasis condition in a laboratory or point-of-care setting [1]. Currently there are two main TEG platforms measuring viscoelasticity properties of whole blood. Both use different mechanisms to assess similar components of the coagulation process. The traditional TEG5000**®** (Haemonetics Corp, USA) system measures the shear elasticity of a coagulating sample using an electrical-mechanical transducer of movement of a torsion wire connected to the suspended pin. Each TEG assay has to be performed individually requiring lengthy preparation and calibrated pipetting [2]. More recently, automation provided by the newer TEG6S**®** (Haemonetics, Illinois, USA) platform eliminates the need for manual pipetting, simplifying and standardising the process. Additionally, unlike the TEG5000 system, the TEG6S enables multiple assays to be performed simultaneously from a single blood sample. The assays include Kaolin TEG, Kaolin TEG with heparinase, Rapid-TEG**®**, and TEG Functional fibrinogen**®** [3]. By superimposing the results of different assays, a detailed clinical picture of haemostasis can be seen in as little as 10 min [4]. The TEG6S measures clot viscoelasticity throughout the coagulation process by using resonance technology, exposing the blood sample to a fixed vibration frequency [5, 6]. Using LED illumination, an infrared detector measures vertical motion of the coagulating blood meniscus. The greater the clot strength, the higher resonant frequencies [7], which is subsequently identified by the detector and converted to a graphical image. The TEG6S can deliver the same quality test results without the complicated test preparation process required when using older TEG5000 platform [6, 8]. The aim of this study was to assess agreement and interchangeability between the TEG6S and TEG5000 analysers.

## Methods

The Austin Health Research and Ethics Committee, Austin Health, Melbourne, Australia, approved this study (number: 05006/2013) November 2014 (chair: Prof. David Taylor) and granted a waiver of participant consent. The study was registered with the Australian New Zealand Clinical Trials Registry (ACTRN12617000062325).

### Study design

We conducted a prospective, observational study at a tertiary metropolitan hospital with a dedicated cardiac and complex hepatobiliary surgical service, including liver transplantation. All participants were recruited at the Austin Hospital, Melbourne, Australia. TEG sampling was conducted by a single skilled operator, over a two-week period in September 2015, between 08 h00 and 18 h00, excluding weekends. Testing only occurred when both TEG devices were operational.

### Participants, inclusion and exclusion criteria

Any adult patient (age > 18 years) admitted to our intensive care unit that required an arterial line and a TEG as part of standard care, was eligible for inclusion. Standard care was defined as the prescribing of a TEG as part of routine diagnostic management to provide the clinician with a point-of-care viscoelastic assessment of the haemostatic process to either detect complex coagulopathies, or for directing rational blood product and coagulation factor therapy. A deliberately heterogeneous sample cohort was selected at random via convenience sampling. In order to assess TEG6S and TEG5000 performance on patients with a range of coagulation states, no exclusion criteria were imposed. Once patients were enrolled, all samples were run simultaneously across two TEG6S and one TEG5000 device. Agreement within a healthy population, and reproducibility of results was the object of a separate investigation.

### Test methods

The primary aim was to determine agreement and interchangeability of the TEG6S and TEG5000 platforms. We used the sampling methodology previously described by Lloyd-Donald et al. [1]. The TEG6S global haemostasis test provides four assays in one multi-channel cartridge, with each assay able to measure various components of the clotting process. We assessed: R time (min), K time (min), alpha angle (degrees), maximum amplitude (mm), and LY30% (%) on the standard citrated kaolin (CK) assay. Each of the TEG devices were calibrated appropriately with biological quality controls. Treating clinicians were blinded to the results of both TEG6S and TEG5000, however operators were neither blinded to either TEG results or clinical information of the patients. Blood was sampled from the patient’s arterial line by a single skilled TEG technician, as previously described [1]. As there is no need for controlled pipetting or prior manipulation of reagents, an unmetered amount of blood (~ 0.5 mL) was removed from the citrated tube and placed into each loaded TEG6S cartridge. All TEG6S assays were performed in automatically loaded microfluidic cartridges. TEG5000 assay sampling was performed manually using recalcification with 20 μl of calcium carbonate solution, and activation by kaolin, as per standard TEG protocol. Patient characteristics and reason for ICU admission was recorded.

### Analysis

“*Interdevice”* agreement was estimated using Lin’s concordance correlation coefficient (Lin’s CC), intraclass correlation coefficient (ICC) and reduced major axis regression (RMAR) [9–12]. We used the same statistical methodology previously described by Wood et al. [13]. RMAR allows disagreement to be separated into fixed and proportional components [14]. The two devices can provide readings that differ by a consistent amount across the magnitude of the readings (fixed bias) or that differ by a changing amount across magnitude (proportional bias) – in the latter case the slope of the reduced major axis regression line will differ from that of the line of perfect agreement (slope = 1) [15]. Power analyses were calculated across all TEG parameters assessed. Due to the absence of pre-existing information regarding anticipated levels of agreement, the appropriate sample size was determined based on pragmatic considerations of having 0.8 power of being able to reject a null hypothesis of moderate agreement in favour of an alternative of excellent agreement for a generic outcome assuming alpha 0.05. Using the method described by Walter et al., wherein, assuming Type I error level alpha of 0.05, two independent rating devices, and the null hypothesis of inter-device agreement of 0.5 to be rejected, the sample size of 22 patients would yield 0.8 power to reliably observe excellent agreement of 0.8 or above [16]. This study recruited 25 patients to account for potential non-evaluable data. No correction for multiplicity of outcomes was made due to the exploratory nature of the study. Statistical analysis was performed using commercial statistical software STATA/IC v.13IC**®** (StataCorp, College Station, TX, USA), using a *p* value of 0.05 to indicate the threshold for statistical significance. Indeterminate or missing data when secondary to true values being lower than what was recordable were assigned a value of zero, and when secondary to true values being higher than recordable were assigned the maximal value for that parameter, or, for the case of TEG5000 maximum amplitude, measured manually from the graphical representation. Missing data due to failed sample analysis from either the TEG6S or TEG5000 were omitted and left blank, as per correlation coefficient calculation standards.

## Results

### Participants

Samples were collected from 25 consecutive adult ICU patients requiring a TEG as part of standard care. The median (range) age was 59 years (23–86). Sixteen (64%) patients were male. Admission ICU diagnosis: post-cardiac surgery (24%), decompensated liver disease (16%), post-liver transplantation (16%), post-general surgery (8%), neurological injury (16%), other critical illness (12%) (Table 1). All patients who required a TEG during the collecting period were included. All samples were taken from patients within 72 h of their hospital admission. There were no patients on therapeutic anticoagulation therapy including novel oral anticoagulants (direct thrombin and factor Xa inhibitors), warfarin, or heparin. There was one failed TEG5000 sample analysis and no violations of the study protocol. The one TEG5000 analysis was missed due to a device error (the cartridge pin dropped prematurely whilst analysing the sample).

**Table 1:** Baseline patient characteristics. Data presented as number (proportion) or median (range)

Male	16 (64%)
Age (years)	59 (23–86)
Admission diagnosis	
Cardiac surgery	6
Cardiac (non-surgical)	2
Liver Disease	4
Acute	1
Chronic	3
Post-liver Transplant	4
Renal failure	2
Neurological	3
Overdose	1
Epilepsy	1
Syncope for investigation	1
Cerebrovascular	1
Other	3
Head and neck infection	1
Addisonian crisis	1
Oncological	1

### Test results

**Fig. 1:**
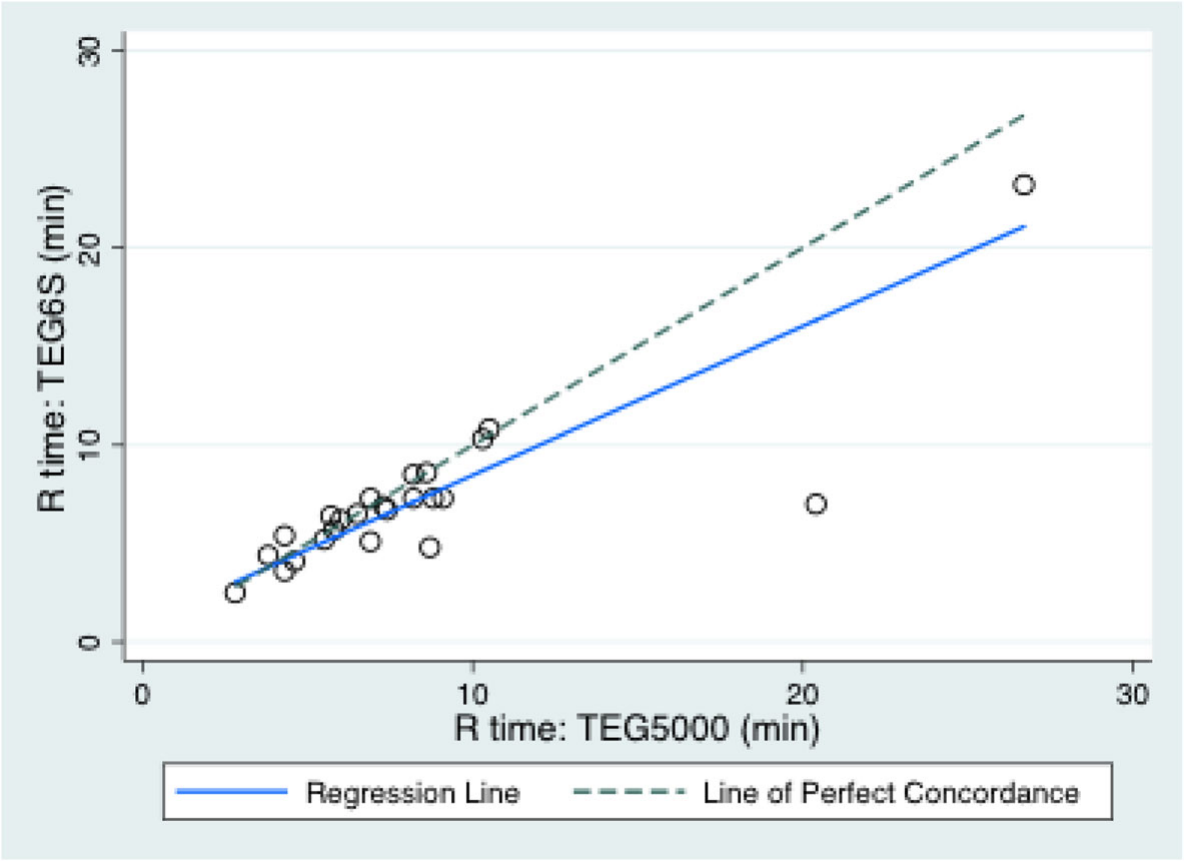
Reaction R time (minutes) between TEG6S and TEG5000 systems

**Fig. 2:**
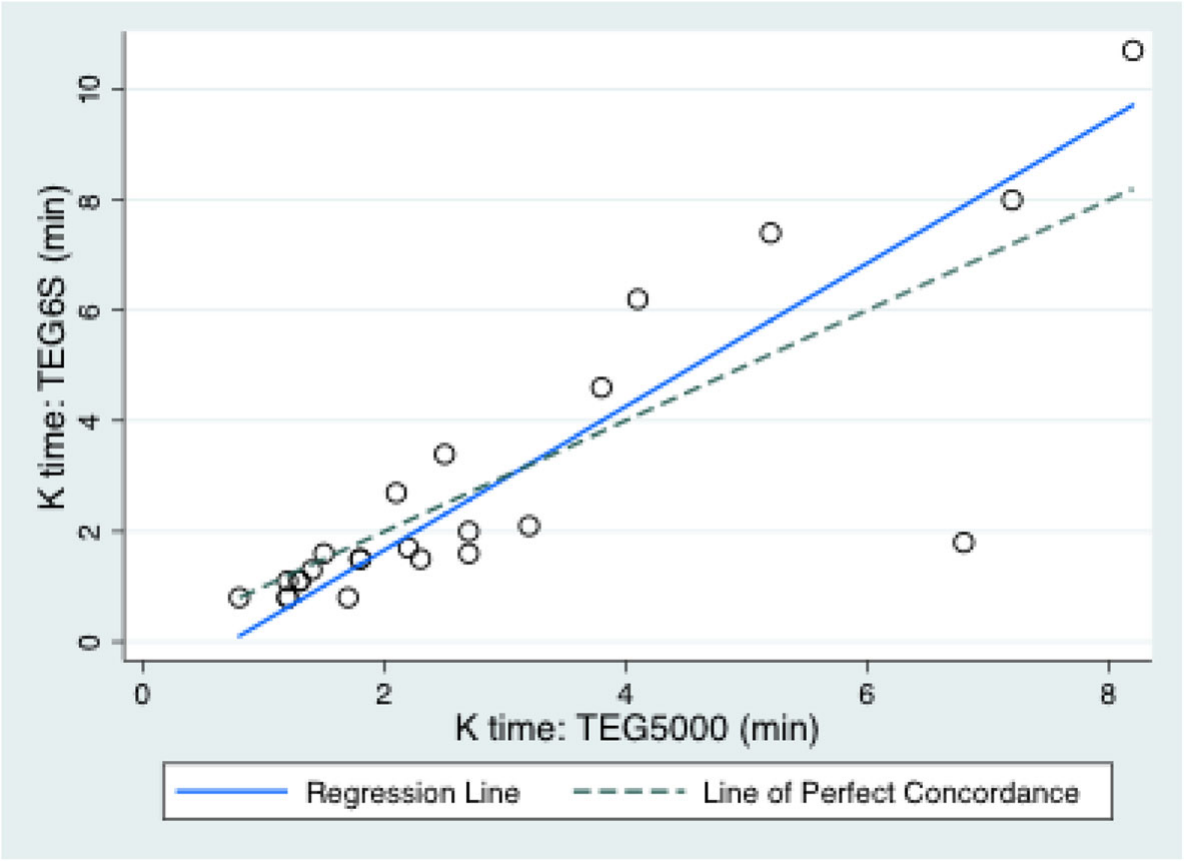
Kinetics K time: (minutes) between TEG6S and TEG5000 systems

**Fig. 3:**
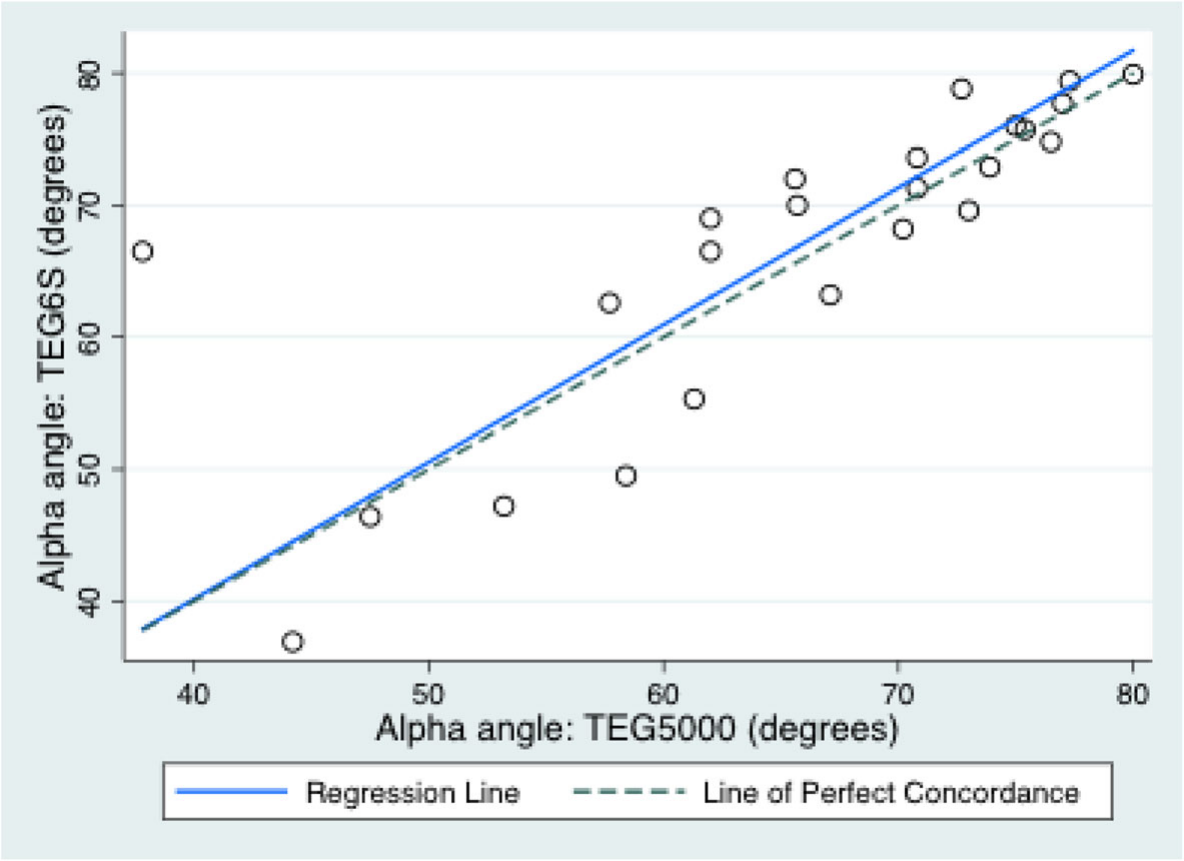
Alpha angle (degrees) between TEG6S and TEG5000 systems

**Fig. 4:**
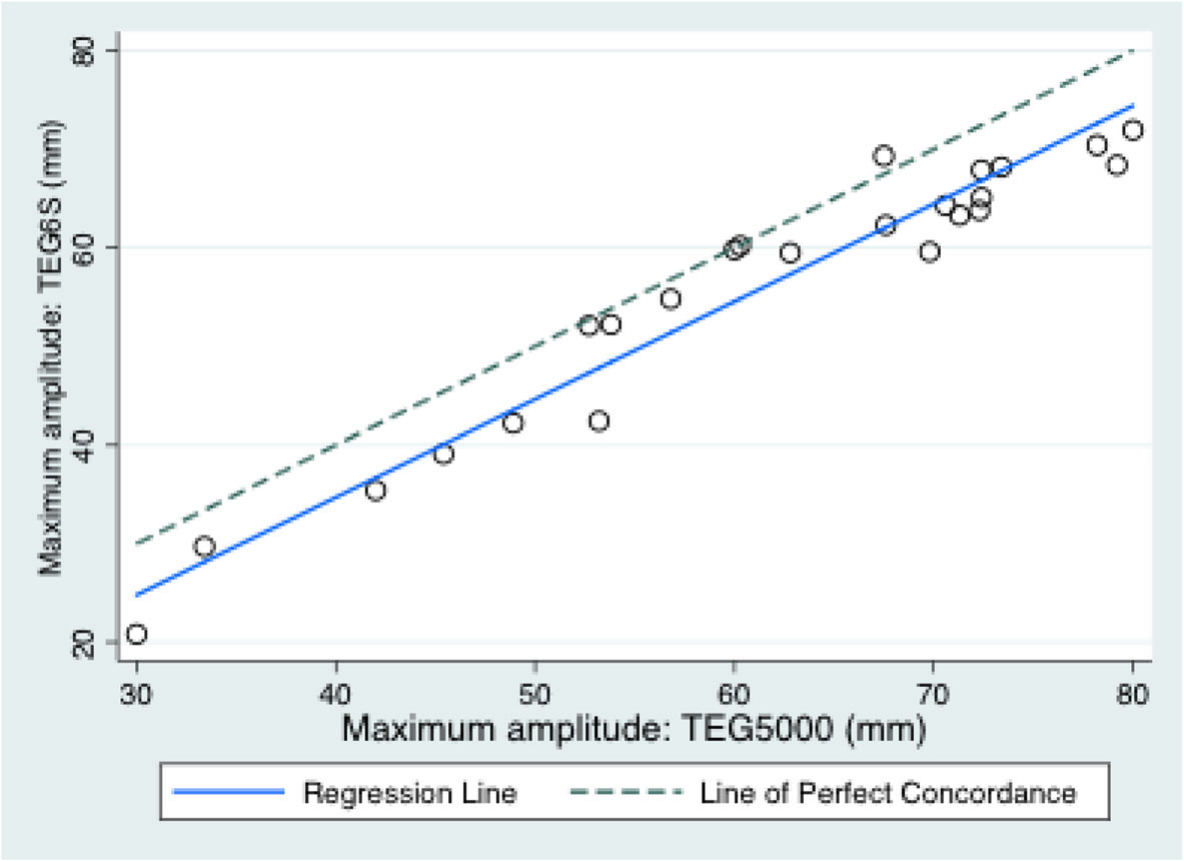
Maximum amplitude (mm) between TEG6S and TEG5000 systems

**Fig. 5:**
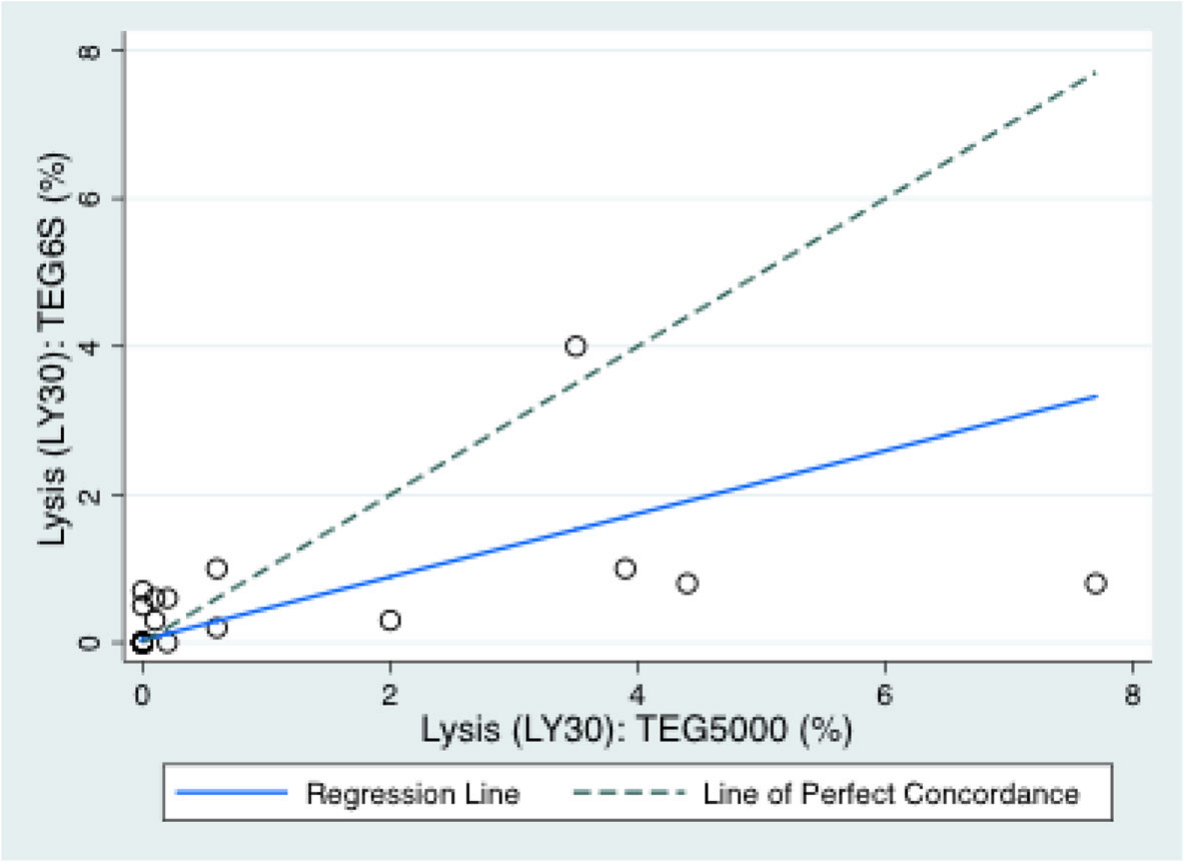
Lysis 30 (%) between TEG6S and TEG5000 systems

The mean (SD) variables measured on the TEG6S and TEG5000 were: R time: 7.3 (4.1) and 8.2 (5.2) minutes; K time: 2.1 (2.5) and 2.8 (2.1) minutes; alpha angle: 67.3 (10.4) and 65.6 (11.2) degrees; MA: 56.2 (13.4) and 61.4 (14.1) millimetres; and LY30%: 0.39 (0.8) and 1.0 (2.0) percent, respectively. The TEG6S and TEG5000 devices generated the following Lin’s CC (95% CI; RMAR slope, intercept): R time: 0.78 (0.64–0.92; 0.76, 0.92); K time: 0.82 (0.69–0.94; 1.30, − 0.93); alpha angle: 0.79 (0.64–0.95; 1.04, − 1.43); MA: 0.90 (0.83–0.96; 0.99, − 5.0); LY30%: 0.34 (0.1–0.58; 0.43, 0.04). Concordance between the TEG6S and TEG5000 platforms is summarised in Table 2 and presented graphically in Figs. 1, 2, 3, 4, and 5, with coefficient of variation (CV) data in Additional file 1 Appendix 1.

**Table 2:** Concordance between the TEG6S and TEG5000 systems

Parameter	Lin’s CC	RMAR Slope	RMAR Intercept	Intraclass correlation coefficient
Reaction time	0.782 (0.640–0.924)	0.755	0.923	0.787
Kinetic time	0.816 (0.690–0.941)	1.298	−0.934	0.823
Alpha angle	0.791 (0.635–0.946)	1.039	−1.429	0.798
Maximum amplitude	0.896 (0.828–0.964)	0.992	−4.977	0.896
LY30%	0.336 (0.097–0.575)	0.427	0.035	0.335

## Discussion

### Key findings

We conducted a prospective observational study determining the interchangeability of the TEG6S and TEG5000 platforms in a cohort of critically ill patients in the intensive care unit of a tertiary hospital. To date, this is the most comprehensive validation study of the TEG6S vs. TEG5000 systems in a critically ill population. We found that the TEG6S and TEG5000 systems were broadly interchangeable. The majority of TEG variables demonstrated almost perfect or substantial agreement, with minimal fixed bias or proportional bias. Exceptions were MA, which demonstrated a fixed, non-proportional bias and LY30%, which demonstrated proportional bias between TEG6S and TEG5000 analysers. Although minor differences in some variables were detected, all variables measured by TEG6S fell within acceptable normal limits. As such, observed differences between TEG6S and TEG5000 were likely clinically insignificant and even the apparent bias in MA (difference 5.2 mm) and difference in LY30% (0.61%) would not likely impact clinical decision-making if within normal limits. This has important implications for use interchangeability of TEG platforms in clinical practice and also for comparing multi-site research programs.

### Relationship to previous literature

One published study has reported the agreement between TEG6S and TEG5000 by assessing a cohort of 300 cardiac surgery patients and 157 healthy controls [8]. Results from the TEG6S were compared to standard reference ranges of the TEG5000 system [8]. Similar to our findings, this study demonstrated a strong correlation between the two systems for the standard haemostasis tests (R *r =* 0.932, MA *r =* 0.972, LY30% *r =* 0.938). However, we used Lin’s CC to assess the degree of agreement between each of the coagulation the variables studied, rather than regression analysis, which assesses correlation [17]. Additionally, the study by Gurbel et al. only compared the agreement of TEG5000 and TEG6S in a normocoagulative cohort (i.e. healthy volunteers and simple cardiac revascularisation). Conversely, we examined the agreement of TEG5000 and TEG6S systems in critically ill patients, including those with sepsis and in chronic liver failure, prone to grossly abnormal and less predictable patterns of coagulation [18–22]. Arguably, in a critically ill population where haemostatic failure and derangements of the coagulation system are common, our findings extend the documented clinical interchangeability of the TEG6S and TEG5000 platforms [22].

### Implications of study findings

Our study findings indicate that the TEG5000 and TEG6S devices are broadly interchangeable in the setting of coagulation monitoring in acutely ill patients. This interchangeability was observed across all major components of the coagulation process. In particular, MA seems to generate the strongest concordance between machines. This has particular clinical relevance as the role thrombus strength, and by extension, maximum amplitude, has in determining the global coagulation state is being recognised as increasingly significant [23, 24]. We also found that despite strong concordance between the MA as measured by the TEG5000 and TEG6S devices, a fixed bias was clearly observed. This finding revealed the TEG6S consistently measures MA higher than that of the TEG5000. To our knowledge, our study is the first to demonstrate this. Whether this finding is clinically significant is impossible to infer from this data alone, however as under-estimation or over-estimation of maximum amplitude may have significant decision-making implications (influencing transfusion requirements), it is an important bias to recognise. Whether the TEG5000, or TEG6S more accurately reflects the true coagulation strength also remains unclear. Whether the TEG5000 or the TEG6S is more closely representative of the true coagulation state requires further research, involving comparison to conventional coagulation tests and clinical outcomes, which goes beyond the scope of this study. Of note, one exception to the close concordance between the two devices occurred when examining LY30%, the parameter measuring fibrinolysis. It is unknown why the concordance between the TEG5000 and TEG6S machines was poor in this parameter and further research is necessitated before LY30% can be used as reliable assessment of fibrinolysis in a clinical setting.

### Strengths and limitations

Our study has several methodological strengths. This is the first study to use rigorous statistical methodology to determine agreement and interchangeability of the TEG6S and TEG5000 platforms. A single skilled operator, blinded to the results, minimised interoperator confounding. All blood samples were taken using the same sampling methodology, in same facility, and in same time-period. Our study was not without limitations. Firstly, our study examines coagulation assessment in a deliberately heterogeneous cohort of intensive care patients, and studies are needed to determine if our findings are applicable to other complex cohorts of patients e.g. cardiac surgery, liver transplantation. However the results of our study reflect agreement and interchangeability of both TEG systems in clinical context of critically ill patients with profound derangements of coagulation tests. This renders this study generalisable to similar centers with similar clinical workloads. Another limitation was the exclusive examination of the citrated kaolin assay, via arterial sampling. Because of this, we are unable to comment as to whether our data is generalisable to venous samples, and are unable to examine agreement across other assay types. However, as this is the most common assay used, and citrate is the most practical storage medium, analysis of the CK assay allowed for the investigation of clinically relevant comparison across both devices. We included a comparatively small sample size, however it was both adequately powered to allow us to complete our study objectives, and is the largest study of its kind in an acutely ill population. Finally, our study was non-interventional, observational research, and subsequently did not have the power to allow authors to compare the diagnostic utility of TEG6S and TEG5000 for clinical outcomes, however, this was not the intention of this study. Conversely, we deliberately aimed to conduct a purely observational study in order to compare and validate a new and existing technology in a controlled, non-interventional setting, in order to provide a complete and minimally biased assessment of an increasingly popular global coagulation assessment tool.

## Conclusions

In critically ill patients, the novel TEG6S platform generates similar and seemingly interchangeable results to the existing TEG5000 model. The majority of TEG variables demonstrated almost perfect or substantial agreement, with minimal fixed bias or proportional bias except for the MA, which demonstrated a fixed non-proportional bias. TEG6S correlates poorly with the TEG5000 for assessing LY30% (clot lysis) and thus is not clinically interchangeable for this purpose. Our findings have important implications for the interchangeability of TEG6S and TEG5000 platforms in clinical practice and in multi-site research programs.

## Additional file

Additional file 1: **Appendix 1**. TEG6S machine 1 and machine 2, and TEG5000 systems coefficient of variation analyses. (DOCX 66 kb)

## Abbreviations

CK: Citrated kaolin, thromboelastography assay
ICC: Intraclass correlation coefficient. Statistical tool used to measure concordance
K time: Clot kinetics, thromboelastography parameter. Measures the time from thrombus amplitude reaching 2 mm until amplitude reaches 20 mm
Lin’s CC: Lin’s concordance coefficient. Statistical tool used to measure concordance
LY30%: Lysis percentage, thromboelastography parameter. Measure of fibrinolysis, represented by degree of clot degeneration after maximum amplitude is reached
MA: Maximum amplitude, thromboelastography parameter. Measures maximum amplitude of the thrombus, being a measure of thrombus strength
R time: Clot reaction time, thromboelastography parameter. Measures the time from the start of analysis until thrombus amplitude reaches 2 mm
RMAR: Reduced major axis regression
TEG: Thromboelastography. Rapid coagulation testing technique providing global assessment of coagulation, using principles of viscoelasticity
TEG5000®: Thromboelastography model 5000, previous model available from Haemonetics Corp, USA
TEG6S®: Thromboelastography model 6S, newest model available from Haemonetics Corp, USA
