# Supplementary material for: Assessment of agreement and interchangeability between the TEG5000 and TEG6S thromboelastography haemostasis analysers: a prospective validation study
Source: BMC Anesthesiol. 2019 Mar 30;19:45. doi: 10.1186/s12871-019-0717-7 (PMC6441230; doi:10.1186/s12871-019-0717-7)
Supplement: Supplementary file 1 — Appendix 1. TEG6S machine 1 and machine 2, and TEG5000 systems coefficient of variation analyses. (DOCX 66 kb) [file 12871_2019_717_MOESM1_ESM.docx]

**APPENDIX**

Appendix 1. TEG6S machine 1 and machine 2, and TEG5000 systems coefficient of variation analyses.

| **Variable** | **TEG6S Machine 1** | **TEG6S Machine 2** | **TEG5000** |
| --- | --- | --- | --- |
| CK Reaction time | 54.23 | 58.94 | 63.47 |
| CK Kinetic time | 97.10 | 97.36 | 72.39 |
| CK Alpha angle | 17.10 | 14.12 | 17.11 |
| CK Maximum amplitude | 24.45 | 23.86 | 22.94 |
| CK LY30% | 179.74 | 213.10 | 201.83 |
| Rapid TEG Reaction time | 97.83 | 93.96 |  |
| Rapid TEG Kinetic time | 270.32 | 119.62 |  |
| Rapid TEG Alpha angle | 17.03 | 17.11 |  |
| Rapid TEG Maximum amplitude | 33.94 | 25.84 |  |
| Rapid TEG LY30% | 482.46 | 227.20 |  |
| Rapid TEG Activated clotting time | 65.11 | 58.54 |  |
| HK Reaction time | 39.88 | 42.10 |  |
| HK Kinetic time | 100.38 | 102.13 |  |
| HK Alpha angle | 16.62 | 14.52 |  |
| HK Maximum amplitude | 23.37 | 23.45 |  |
| Fib Maximum amplitude | 62.43 | 61.04 |  |
| Functional fibrinogen level | 60.15 | 58.38 |  |
